# Supplementary material for: Internet gaming disorder associated with depression among Thai medical students: A university-based cross-sectional study
Source: PLoS One. 2025 Jul 11;20(7):e0327820. doi: 10.1371/journal.pone.0327820 (PMC12250579; doi:10.1371/journal.pone.0327820)
Supplement: Attachment — Submitted filename: Response to Reviewers.pdf [file pone.0327820.s004.pdf]

Response to Reviewers

Reviewer #1: Good topic to discuss, but needs some clarification

1- what types of physical illness, and could that affects results

Response: Thanks for recommendation. We've added these information as "Among those with physical illnesses, the most common conditions reported were allergies (8.0%), thyroid disease (0.8%), asthma (0.6%), dyspepsia (0.3%), and migraines (0.3%)."

2- you didn't discuss the frequency of Internet gaming, just yes or no, though this may affect results

Response: Thanks for recommendation. We've added these limitation as "A previous study found a strong correlation between the amount of time spent playing online games and the development of depressive symptoms. In particular, playing for more than five hours a day was associated with an increased risk of developing depression [8]. However, this study did not explore the characteristics or patterns of gameplay, which limits the depth of its findings and their applicability in creating specific guidelines for the prevention of IGD. Future studies could provide more detailed insights into gaming behaviors, including the types of games played, the frequency and duration of gaming sessions, and the psychological factors that influence these habits."

3- alcohol and smoking must be clarified more like duration and frequency per day

Response: Thank you for the recommendation. This is a limitation of our study.

4- manuscript needs English proof

Response: Thanks for recommendation. The English of this revised manuscript was proofread/edited by the Office of International Affairs, Faculty of Medicine, Prince of Songkla University.

Finally, thanks to your suggestions, our manuscript has become more scientifically valuable and elegant. We sincerely hope to receive your kindness again and forever.

Reviewer #2:

General comments.

Any study that involves medical students is always of interest especially those that may affect their practice as clinicians in less than no time. The Authors are commended for conceptualizing the study and for their efforts in ensuring that their research work is published. I am of the opinion that the manuscript has merit but may require revision before it could be accepted for publication.

Response: Thanks for your kindness.

Authors should take note of the following observations:

1. Title of study: It appears as if Authors were out to establish a relationship between Internet Gaming Disorder (IGD) and symptoms of depression. This was very evident in the title of the study, the approach to statistical analysis and in the recommendations. If this approach should stand, the inclusion of a conceptual framework will be very essential.

Response: Thanks for your feedback.

2. Conclusion and Recommendation: Authors recommended the concurrent treatment for both IGD and depression and development of healthier coping mechanisms. This recommendation is rather far reaching. In a study involving future clinicians, the focus ought to have been preventive. Thus measures like how to improve the mental health of the students, ensure good interactions between the students and the Faculty members and the early identification of symptoms of depression and IGD ought to have been of priority. Authors should also indicate the implications of study findings.

Response: Thank you for pointing out these issues. We've added these issue according to discussion "Moreover, creating a safe and supportive medical school learning environment, along with fostering good relationships between medical students and faculty members, is crucial. Ensuring open communication, promoting a culture of empathy, and encouraging understanding within medical schools can significantly contribute to the well-being of students. This approach can enhance their overall academic experience and resilience in the face of challenges, ultimately helping to reduce mental health problems among medical students [16, 26]."

3. Sample size determination: A sample size formula was applied in determining the sample size. Authors went ahead to indicate a response rate. This (the response rate) may not be necessary since this was not a total population study.

Response: Thank you for pointing out these issues. We've removed "the response rate".

4. Sampling technique: Authors indicated that a convenience sampling approach was employed in the study. An online questionnaire was used. How did the Authors ensure that the exclusion criteria were adhered to?

Response: Thank you for pointing out these issues. We've revised as suggestion as" The researcher utilized personal contacts to obtain permission and provide information about the study to the administrators of the official social media accounts used by medical students. This approach ensured that the sample consisted exclusively of medical student members, facilitating targeted recruitment while maintaining relevance to the study's focus."

5. The study limitation should include the method used in recruiting respondents for the study. The results may not be a true representation of the issues at stake based on the sampling technique.

Response: We have added this suggested issue as the limitation. "In addition, there is the potential for sample bias, with medical students experiencing high levels of depression or IGD either avoiding participation or being overly represented due to a heightened interest in the survey. This could distort the findings, leading to an inaccurate estimate of the true prevalence of these conditions."

6. Study instrument: Authors should provide a reference for the questionnaires. A reference should also be provided for the cut off of 9 used for Patient Health Questionnaire. What does the Authors mean that, 'For this study, the IGD scale 9 exhibited greater internal consistency with Cronbach's alpha coefficient of 0.71.' Same comment was made for Patient Health Questionnaire.

Thank you for pointing out these issues. The cutoff score of 9 used for the Patient Health Questionnaire is referenced in Reference 19. In addition, we have also cited Reference 19 accordingly in the results section.

The statement "For this study, the IGD scale 9 exhibited greater internal consistency with a Cronbach's alpha coefficient of 0.71" represents the reliability calculation for the instrument based on the data from this study. However, we decided to omit this information to avoid potential confusion for readers.

7. Medical education in Thailand: A brief description of the training of medical students in Thailand will be helpful. For example in table 1, categories like 'Pre-clinic' and 'Clinic' were indicated. What do they mean? How many years are spent in Thailand in studying Medicine?

Response: Thank you for pointing out these issues. We have added this suggested issue as "In the context of medical education in Thailand, the curriculum is divided into two phases. The first three years, known as the pre-clinical phase, focus on acquiring basic scientific knowledge in subjects like anatomy, physiology, and biochemistry. The final three years, referred to as the clinical phase, shift the focus towards practical application. Students in this phase engage in both theoretical coursework and hands-on patient care under the supervision of experienced clinicians."

8. This statement should be clarified; 'The finding that there was no significant difference in symptoms of depression between medical students with or without IGD….' Which table showed this result?

Response: Thank you for pointing out these issues. We have showed the comparing as in Figure 3. And we have added more information as "(p-value > 0.05: range from 0.164 to 1)"

9. Statistical analysis: The age of the students is expected to be normally distributed and should be represented using mean and standard deviation since it is a continuous variable.

Response: Thank you for pointing out these issues. Our study surveyed medical students from the first to the sixth year. The Shapiro-Wilk normality test produced a p-value of <0.001, confirming that the age data were not normally distributed.

For a study that included a binary logistic regression analysis, adjusted odds ratio and 95% confidence interval ought to have been used to show the predictors of the outcome measure of the study instead of p value in the abstract. Why was table 2 and 3 separated?

Response: Thank you for highlighting these points. Table 2 presents the univariate analysis, which typically does not include adjusted odds ratios, as it focuses on the individual, crude relationships between each variable and the outcome. The purpose of this table is to offer an initial overview of potential associations without adjusting for confounding factors.

In contrast, Table 3 (the multivariate analysis) presents adjusted odds ratios, which are essential for understanding the independent effect of each variable while controlling for other factors in the model.

Authors indicated that a p value of <0.2 was used in including variables into the logistic regression model after bivariate analysis; was this adhered to?

Response: Thank you for highlighting these points. We have explained more as "The significant variables identified included gender, presence of physical illness, history of substance use, current stress, and depression (Table 2)."

The column for IGD, 'Yes' should come first in table 2.

Response: Thank you for highlighting these points. We have revised as 'Yes' comes first.

How were the two non-responses for gender taken care in bivariate and multivariate analysis in tables 2 and 3? This should be explained*?*

Response: The 2 cases are missing values and therefore were not included in the analysis of the gender variable. This has been added in the "Association" section as "Due to only two non-responses for gender, these were excluded from both the bivariate and multivariate analyses."

A proportion of 30.8% should be reported as less than one third.

Response: Thank you for highlighting these points. We have revised as suggestion "Less than one-third of the participants (30.8%) reported a history of alcohol use, primarily involving social drinking."

10. Comments related to studies ought to have more than one reference. Authors should avoid the use of Arabic numerals in the body of the manuscript. This may conflict with the reference numbers. Include Thailand as a key word. Instead of signs of depression, use symptoms of depression.

Response: Thank you for pointing out these issues. We have added more than one reference such as "A prior study reported the prevalence of depression among Thai medical students ranged from 11.1% to 15.9%, indicating a significant mental health concern [16, 17]."

We have instead of signs of depression, use symptoms of depression as "symptoms of depression; indicated by a PHQ-9 score of 9 or higher."

11. Questionnaire 1 as indicated by the Authors included the CGPA. This was not included in the result section. This should be clarified. If there was a variable like 'Perceived academic performance' it could have also been useful to see its relationship with IGD and symptoms of depression. The categories for depression in tables 2 and 3 should be renamed. Depression symptoms, 'Yes' and 'No' will be more appropriate.

Response: Thank you for highlighting these issues. We have added additional information as suggested, including "GPA," and revised the categories for depression in Table 2.

12. Authors should review the manuscript critically for minor errors of grammar.

    Response: Thanks for recommendation. The English of this revised manuscript was proofread/edited by the Office of International Affairs, Faculty of Medicine, Prince of Songkla University.

    Finally, thanks to your suggestions, our manuscript has become more scientifically valuable and elegant. We sincerely hope to receive your kindness again and forever.
