# Supplementary material for: Internet gaming disorder associated with depression among Thai medical students: A university-based cross-sectional study
Source: PLoS One. 2025 Jul 11;20(7):e0327820. doi: 10.1371/journal.pone.0327820 (PMC12250579; doi:10.1371/journal.pone.0327820)
Supplement: Attachment — Submitted filename: response to reviewer V3.pdf [file pone.0327820.s006.pdf]

Review comments

General comments

I commend the Authors for the good review of the manuscript. I am also of the opinion that certain aspects of the manuscript may require another revision before the manuscript could be accepted for publication.

Response: Thanks for your kind recommendation that make our revised manuscript scientifically.

Authors should take note of the following observations:

1. **Journal guidelines.** Authors should adhere to Journal guidelines in presenting the manuscript including the abstract. Authors should refer to the website of the Journal.

Response: We have revised the manuscript pattern as a suggestion.

2. **Abstract.** Authors should delete the Arabic numerals used in listing the number of questionnaires used in the study. Do the same in the body of the manuscript. In the conclusion, Authors should focus on preventive measures instead of treatment options. Some of the results in the abstract were not included in the results section. Thailand should be included as key words.

Response: We have deleted the Arabic numerals used in listing the number of questionnaires used in the study, and revised the conclusion as "Medical schools should establish early detection systems to identify these challenges. By prioritizing prevention strategies for both IGD and depression, medical schools can provide students with the tools to develop healthier coping mechanisms. Encouraging gaming alternatives can significantly enhance their overall well-being and mental health." Additionally, we have added "Thailand" in the part of key words. Thanks for the suggestion.

3. **Statistical analysis.** In line 213, Authors stated that '*variables with a p value of <0.2 from the univariate analysis were selected for inclusion …….*'. Authors should change univariate to bivariate.

Response: We have revised it as suggestion.

In line 222, Authors stated that '*we incorporated significant variables into the multivariate logistic regression model…..*'. The two statements are not in tandem. I align myself with the first statement as corrected. Why were 'physical illness' 'history of substance use' and 'presence of current life stress' not included in the logistic regression model? A revision is required.

Response: We have revised it as suggestion.

4. **Conceptual framework**. I am of the opinion that based on the title of the study, a conceptual framework may be required to solidify the study concept. Adhering to comment 3

above may establish the relevance of the title and not that the analysis was based on being selective.

Response: We have added in line 216-219 "Significant variables were included in a multivariate logistic regression model, with multicollinearity evaluated using the Variance Inflation Factor (VIF). In the final model, variables with a p-value less than 0.05 were considered significantly associated with the outcome."

Introduction. Change 'rises' in line 75. A meta-analysis could not have been done on medical students.

Response: We have revised as suggestion "In Thailand, the prevalence of IGD among students in a rural community school was 5.4% [2], while among university students, it was 14.3% [3]. A meta-analysis indicated that the pooled prevalence of IGD among medical students from various countries was 6.2%, approximately double the rate observed in the general population"

Line 85, should have more than one reference since it was based on recent studies.

Response: We have added new reference (no 9).

Line 104, Were more than one ethics committee involved in approving the study?

Response: *Thanks for your mention. Both Hat Yai Hospital Medical Education Center and Yala Hospital Medical Education Center are administrated by the Faculty of Medicine at Prince of Songkla University. This means they must utilize the same ethics committee for any research.*

Considering the sensitive nature of the topics being studied—specifically the prevalence of Internet Gaming Disorder and depression—our ethics committee has expressed concerns and has requested a revision in the way the institutions are described in our study. Therefore, we have been asked to change the wording from "the Faculty of Medicine, Prince of Songkla University, Hat Yai Hospital Medical Education Center, and Yala Hospital Medical Education Center" to "one Faculty of Medicine, and two Medical Education Centers in southern Thailand" to maintain confidentiality and ensure sensitivity.

We kindly request your approval to comply with this revision and ensure that we align with the ethics committee's requirements.

Thank you very much for your understanding and support. Please let us know if you require any further information or clarification.

5. **All acronyms** used in tables should be explained as foot notes

Response: We have added all meanings of acronyms used in footnotes.

Finally, thanks to your suggestions, our manuscript has become more scientifically valuable and elegant. We sincerely hope to receive your kindness again and forever.
