# Supplementary material for: Internet gaming disorder associated with depression among Thai medical students: A university-based cross-sectional study
Source: PLoS One. 2025 Jul 11;20(7):e0327820. doi: 10.1371/journal.pone.0327820 (PMC12250579; doi:10.1371/journal.pone.0327820)
Supplement: Attachment — Submitted filename: Response to reviewer V4.pdf [file pone.0327820.s007.pdf]

Point to point response

Review comments

I remain grateful to the Authors for the good review of the manuscript. My intention was that the last review should be the last revision for the manuscript signaling the recommendation for the acceptance of the manuscript for publication.

However, a careful review of the manuscript indicated that there are some contradictions in the manuscript which should be reconciled before the manuscript could be accepted for publication.

Response: Thank you for your kindness in giving us the opportunity to correct the information before accepting our manuscript.

1. In lines 147 – 149 of the manuscript indicated how the total depressive score of respondents should be categorized. "Total scores range from 0 to 27, with interpretations as follows: 0-4 (no or minimal depression), 5-9 (mild depression), 10-14 (moderate depression), 15-19 (moderately severe depression), and 20-27 (severe depression)." Commencing from the same line 149 Authors stated that 'a cut off score of nine or higher is considered indicative of depression.' No reference was provided for the use of cut off score of 9. This should be clarified.
   Response: We have coded reference 20,21.

2. The report in table is different from that in table 2 with regards to the sample size of the study. In table 1, the total number of respondents was 634. This was indicated in line 163. The total number as indicated in table 2 is 510 except gender. This should be reconciled.

   Response: We surveyed a total of 634 medical students. However, only 510 students reported engaging in game playing (as indicated by the "N" in Table 2, which reflects only those who play games). Among these 510 students, 2 did not report their gender, resulting in missing gender data (as we described in line 220). Therefore, we have added this clarification as in line 212-220.

3. Authors should refer to lines 206 -210. However, in line 217, Authors indicated the use of significant variables as basis for inclusion into the logistic regression model after bivariate analysis. These comments as indicated make table 3 very confusing. Authors should state the basis for inclusion of variables into the regression model after applying the Chi square test.

   Response: We have corrected and added this clarification as show in line 227-229.

4. Authors should include convenience sampling in the abstract.

   Response: We have added the convenience sampling in the abstract as "Participants were recruited using convenience sampling……."

5. Authors should use Roman numerals instead of Arabic in list the study instrument in the body of the manuscript. (Lines 135, 138 and 144).

Response: We have changed Roman numerals instead of Arabic in list the study instrument. Thank you for your kindness.
