# Supplementary material for: Internet gaming disorder associated with depression among Thai medical students: A university-based cross-sectional study
Source: PLoS One. 2025 Jul 11;20(7):e0327820. doi: 10.1371/journal.pone.0327820 (PMC12250579; doi:10.1371/journal.pone.0327820)
Supplement: Attachment — Submitted filename: Review comments.docx [file pone.0327820.s001.docx]

**Review comments**

**General comments.**

Any study that involves medical students is always of interest especially those that may affect their practice as clinicians in less than no time. The Authors are commended for conceptualizing the study and for their efforts in ensuring that their research work is published. I am of the opinion that the manuscript has merit but may require revision before it could be accepted for publication.

Authors should take note of the following observations:

1. Title of study: It appears as if Authors were out to establish a relationship between Internet Gaming Disorder (IGD) and symptoms of depression. This was very evident in the title of the study, the approach to statistical analysis and in the recommendations. If this approach should stand, the inclusion of a conceptual framework will be very essential.
2. Conclusion and Recommendation: Authors recommended the concurrent treatment for both IGD and depression and development of healthier coping mechanisms. This recommendation is rather far reaching. In a study involving future clinicians, the focus ought to have been preventive. Thus measures like how to improve the mental health of the students, ensure good interactions between the students and the Faculty members and the early identification of symptoms of depression and IGD ought to have been of priority. Authors should also indicate the implications of study findings.
3. Sample size determination: A sample size formula was applied in determining the sample size. Authors went ahead to indicate a response rate. This (the response rate) may not be necessary since this was not a total population study.
4. Sampling technique: Authors indicated that a convenience sampling approach was employed in the study. An online questionnaire was used. How did the Authors ensure that the exclusion criteria were adhered to?
5. The study limitation should include the method used in recruiting respondents for the study. The results may not been a true representation of the issues at stake based on the sampling technique.
6. Study instrument: Authors should provide a reference for the questionnaires. A reference should also be provided for the cut off of 9 used for Patient Health Questionnaire. What does the Authors mean that, ‘For this study, the IGD scale 9 exhibited greater internal consistency with Cronbach’s alpha coefficient of 0.71.’ Same comment was made for Patient Health Questionnaire.
7. Medical education in Thailand: A brief description of the training of medical students in Thailand will be helpful. For example in table 1, categories like ‘Pre-clinic’ and ‘Clinic’ were indicated. What do they mean? How many years are spent in Thailand in studying Medicine?
8. This statement should be clarified; ‘The finding that there was no significant difference in symptoms of depression between medical students with or without IGD….’ Which table showed this result?
9. Statistical analysis: The age of the students is expected to be normally distributed and should be represented using mean and standard deviation since it is a continuous variable. For a study that included a binary logistic regression analysis, adjusted odds ratio and 95% confidence interval ought to have been used to show the predictors of the outcome measure of the study instead of p value in the abstract. Why was table 2 and 3 separated? Authors indicated that a p value of <0.2 was used in including variables into the logistic regression model after bivariate analysis; was this adhered to? The column for IGD, ‘Yes’ should come first in table 2. How were the two non-responses for gender taken care in bivariate and multivariate analysis in tables 2 and 3? This should be explained? A proportion of 30.8% should be reported as less than one third. Authors should also include a correlation analysis between IGD and depression scores.
10. Comments related to studies ought to have more than one reference. Authors should avoid the use of Arabic numerals in the body of the manuscript. This may conflict with the reference numbers. Include Thailand as a key word. Instead of signs of depression, use symptoms of depression. In line 75, review the use of ‘..rises to 14.3%’.
11. Questionnaire 1 as indicated by the Authors included the CGPA. This was not included in the result section. This should be clarified. If there was a variable like ‘Perceived academic performance’ it could have also been useful to see its relationship with IGD and symptoms of depression. The categories for depression in tables 2 and 3 should be renamed. Depression symptoms, ‘Yes’ and ‘No’ will be more appropriate.
12. Authors should review the manuscript critically for minor errors of grammar.
