# Supplementary material for: Internet gaming disorder associated with depression among Thai medical students: A university-based cross-sectional study
Source: PLoS One. 2025 Jul 11;20(7):e0327820. doi: 10.1371/journal.pone.0327820 (PMC12250579; doi:10.1371/journal.pone.0327820)
Supplement: Attachment — Submitted filename: Review comments.docx [file pone.0327820.s003.docx]

**Review comments**

**General comments**

I commend the Authors for the good review of the manuscript. I am also of the opinion that certain aspects of the manuscript may require another revision before the manuscript could be accepted for publication.

Authors should take note of the following observations:

1. **Journal guidelines.** Authors should adhere to Journal guidelines in presenting the manuscript including the abstract. Authors should refer to the website of the Journal.
2. **Abstract.** Authors should delete the Arabic numerals used in listing the number of questionnaires used in the study. Do the same in the body of the manuscript. In the conclusion, Authors should focus on preventive measures instead of treatment options. Some of the results in the abstract were not included in the results section. Thailand should be included as key words.
3. Statistical analysis. In line 213, Authors stated that ‘*variables with a p value of <0.2 from the univariate analysis were selected for inclusion …….*’. Authors should change univariate to bivariate. In line 222, Authors stated that ‘*we incorporated significant variables into the multivariate logistic regression model…..’.* The two statements are not in tandem. I align myself with the first statement as corrected. Why were ‘physical illness’ ‘history of substance use’ and ‘presence of current life stress’ not included in the logistic regression model? A revision is required.
4. Conceptual framework. I am of the opinion that based on the title of the study, a conceptual framework may be required to solidify the study concept. Adhering to comment 3 above may establish the relevance of the title and not that the analysis was based on being selective.
5. Introduction. Change ‘rises’ in line 75. A meta-analysis could not have been done on medical students. Line 85, should have more than one reference since it was based on recent studies. Line 104, Were more than one ethics committee involved in approving the study?
6. All acronyms used in tables should be explained as foot notes
