# Supplementary material for: Internet gaming disorder associated with depression among Thai medical students: A university-based cross-sectional study
Source: PLoS One. 2025 Jul 11;20(7):e0327820. doi: 10.1371/journal.pone.0327820 (PMC12250579; doi:10.1371/journal.pone.0327820)
Supplement: Attachment — Submitted filename: Review comments.docx [file pone.0327820.s005.docx]

**Review comments**

I remain grateful to the Authors for the good review of the manuscript. My intention was that the last review should be the last revision for the manuscript signaling the recommendation for the acceptance of the manuscript for publication.

However, a careful review of the manuscript indicated that there are some contradictions in the manuscript which should be reconciled before the manuscript could be accepted for publication.

1. In lines 147 – 149 of the manuscript indicated how the total depressive score of respondents should be categorized. “**Total scores range from 0 to 27, with interpretations as follows: 0-4 (no or minimal depression), 5-9 (mild depression), 10-14 (moderate depression), 15-19 (moderately severe depression), and 20-27 (severe depression).”**

Commencing from the same line 149 Authors stated that ‘**a cut off score of nine or higher is considered indicative of depression.**’ No reference was provided for the use of cut off score of 9. This should be clarified.

2. The report in table is different from that in table 2 with regards to the sample size of the study. In table 1, the total number of respondents was 634. This was indicated in line 163. The total number as indicated in table 2 is 510 except gender. This should be reconciled.
3. Authors should refer to lines 206 -210. However, in line 217, Authors indicated the use of significant variables as basis for inclusion into the logistic regression model after bivariate analysis. These comments as indicated make table 3 very confusing. Authors should state the basis for inclusion of variables into the regression model after applying the Chi square test.
4. Authors should include convenience sampling in the abstract.
5. Authors should use Roman numerals instead of Arabic in list the study instrument in the body of the manuscript. (Lines 135, 138 and 144).
